# Regional Debility in Riggs’ Disease

**Published:** 1917-07

**Authors:** M. H. Cazier

**Affiliations:** New York, N. Y.


### REGIONAL DEBILITY IN RIGGS' DISEASE

BY M. H. CAZIER, M.D., NEW YORK, N. Y.

Assuming that the dominant thought of the dental profession is reflected in the current literature, we may reduce to concreteness present-day conceptions of the pathology of Riggs' disease by the phrase, "*inflammation* due primarily to *irritation*," or "*infection* due primarily to a specific microorganism," and assume that the practice of its votaries consists of efforts to remove the mechanical irritation or to kill the offending germ, adding such agencies as are supposed to reduce or control the inflammatory process.

The appearance of this paper upon your program is due to the fact that its author entertains beliefs which are at absolute variance with both of these postulates.

First of all, my belief is that the real condition is not an inflammatory but a degenerative process, and if any inflammation attends it is incidental and unimportant; and here the entire issue is joined, for if we show this to be a fact, then the remaining postulate and the practice built thereon must be modified to meet the indications of the newer light.

Among those contending that the tissues are inflamed, one writer gives the following reasons: "The parts bleed on slightest touch"; another, "Pus is welling up around the tooth"; a third, "The gums are swollen and of an unnatural color." Translated, then, the word inflammation must mean *any local condition which is abnormal.* This is a serious error, and must be corrected if we are to reach rational views and rational and successful practice.

Speaking generally, lesions of the body may be divided into two groups, sthenic and asthenic—an old classification, but somewhat instructive.

The sthenic comprises those lesions wherein nature is making active efforts to repair an injury or to resist invasion. This is only possible when the tissues are of approximately normal vitality. This is the inflammatory type.

The asthenic lesion is represented by the minus sign, the group of lesions occurring in parts where nutrition is below par, meaning that the metabolic process is on "low speed." The condition may be local and due to inadequate nutritive supply, caused by local disturbance of circulation; or it may be general and due to constitutional ailments, or it may partake of both causes, as is seen in bedsores, ulcers of the pedal extremities, senile gangrene, and other degenerative conditions.

It must be apparent that ability to distinguish the difference and to recognize its importance is essential as a guide to appropriate practice.

In the sthenic type of lesion the forces of the organism are already at work, and are vigorous enough for the task. Our duty then, is largely to remove the excitant, and observe the phenomena; we have simply nature's process of repairing injuries and healing wounds; it is metabolism on "high speed," in other words *inflammation.* It frequently requires direction or control, but rarely if ever should it be subdued.

In Riggs' disease the lesion is asthenic. and due to inadequate arterial supply with passive venous engorgement—regional debility. This is the case even when the general vital conditions are normal, and the first duty of the surgeon is to restore the vital equilibrium.

It is a well-established fact in histology that the alveolar process is nourished by arterial spurs given off mainly at right angles from the principal arteries of the jaws, and that these spurs, ramifying in the cancellous alveoli, breaking up into capillaries, and returning as veins, and therefore end-organs in bony substance.

The very nature, therefore, of the arterial mechanism carrying the blood supply renders nutrition precarious, and is perhaps never adequate in the majority of individuals, becoming markedly inadequate with the intervention of pregnancy or disease, or when advancing years render the arterial system less resilient, and consequently less efficient. This deficiency manifests itself in the senile degeneration of the alveoli, and if uncomplicated by irritation, results in simple atrophy, the parts shrinking in contour but retaining continuity.

With the advent of irritation from malocclusion or other causes, a solution of continuity occurs, and the waste from the resulting lesion forms the deposits, and affords infection the opportunity of becoming the most prominent symptom and impressing the nosology, unhappily, with the name "pyorrhea."

The alveolar process has long been regarded as a "transitory member"; it is so simply and solely because nutrition fails. It has been pointed out in the literature for generations that the process undergoes senile dissolution the first of any tissue of the body, but until very recent time no practical application has ever been made of the fact. It was looked upon as though, like the movements of the planets or the tides of the sea, it were entirely beyond human control; and although this structure is admittedly the essential seat of Riggs' disease, the fact of its habitual degeneration has not until now been recognized as the basic pathological element.

The fact that blood supply can easily be made adequate, that metabolism is encouraged by massage, that supplying these results is a checking of the waste, that when this is accompanied by proper treatment recovery follows, and that the vital resistance so established resists bacterial infection, are now among the well-attested facts of clinical experience. Scores of practitioners on the Atlantic seaboard, with hundreds of patients, witness the success of treatment when the necessary but simple technic is carried out.

The author made a series of studies on cadavers confirmatory of the admitted fact of the senile changes in the alveolar process. The important fact disclosed was that changes in the bone precede the external symptoms. Evident signs of necrosis in spots at the alveolar crest were noted before infection, recession, or deposit had appeared. These facts, together with previous studies of other and more competent observers, place regional debility first among the causes of Riggs' disease, and place irritation, infection, and traumatic injuries in the category of contributory causes.

Regional debility of the investing structure of the teeth affords the condition which accentuates the effect of irritation and invites infection. It is often merely due to the local causes above enumerated, and is to be overcome by local measures adapted to cause a "rerenewal of life" in the part. The condition is frequently aggravated by systemic defects, but it is beyond the province of this paper to discuss that phase of the subject. This has led some authors into an attempt to show as many varieties of this local malady as there are forms of systemic ailments—which can have little other effect than to confuse and confound.

### TREATMENT

The author, for more than thirty years, has treated lesions due to regional debility by inducing arterial hyperemia, with signal success. In rather recent years the practice has been popularized in the literature by Professor Bier of the University of Bonn. The most common method of Bier is to thin the air by suction, reducing the atmospheric pressure, which in turn gives fuller sway to the arterial pressure. The venous radicals are unloaded and arterial hyperemia results. The suction apparatus may easily be used to aspirate sinuses about the teeth, and also may be adapted to produce massage.

Treatment of ailments addressed to secondary causes very frequently dazzle and mislead. Such efforts may modify symptoms and results in improvement, but recovery is reached only in so far as we reach and nullify the continuing fundamental cause.

Whatever methods may be favored by individual practitioners, directed to the removal of secondary causes, irritation or infection, we must realize that to reach real recovery we must ever depend upon the *vis medicatrix naturae,* and so direct the local treatment, as well as the diet and the regimen of our patient, that infection in the mouth shall be met by an overwhelming vital resistance. This is immunity, and it resides in the germicidal power of the blood and the antibodies, and is the protective principle which has saved the human race from extinction through all the ages of the past.

In the purely local treatment, the removal of deposits by operative procedure, it is perhaps possible to attain to such a degree of expertness as enables the operator to proceed in the deeper sinuses, where live and dead tissue is intermingled, blinded by the flood of venous blood, inhibited by the pain the patient suffers, and to remove the offending substance with a minimum of damage to live tissue. But this is rare ability and can minister only to the few, and even with this extra development of operative skill, the author would suggest a reduction of the danger to live tissues, a lessening of the difficulties which beset the procedure, by the employment of such circumspection as the general surgeon uses when he prepares the patient and the field for operation.

This means that the debility of the tissues must be replaced by vital energy, that a line of demarcation shall be established as a guide to the operator, that venous engorgement shall disappear, that tumefied gum tissue due to stasis shall give place to normal, and withal, that a beginning inflammation of live tissue, involving pericemental membrane and bone, evidenced by swelling and exudation, aided by aspiration, shall evict foreign elements wherever attached to structures capable of this reaction. By this means the operation is greatly simplified, and made safe and efficient in the hands of the general practitioner, who after all must minister to the multitude, and in whose hands treatment heretofore has been disappointing.

Reducing the indications to a program this treatment means the postponement of instrumentation, except the removal of extra offending tartar, making it the last thing to be done. First, daily drainage, hyperemia, and massage should be instituted; second, and after a week or ten days, when vital conditions will support reaction, treatment of the walls of the sinus by instilling into the pocket an acidulous solution, every fifth day.* This causes reaction, swelling of the live parts and shrinking of the dead, and besides it deals death to microorganisms, and softens somewhat the deposit. The swelling of the peridental membrane dislodges deposits, the temporary irritation of the marginal surface of the bone stimulates osseous development, sinuses grow smaller, vital and dead tissue is put in contrast, so that "velvet" and "sandpaper" are easily differentiated, and the final scaling and polishing of the necks can be done under more favorable conditions to both operator and patient, and should be made as complete as skill can accomplish.

---

* The author employs a 25 per cent. solution of c.p. sulfuric acid, in 65 per cent. alcohol, to which is added 2.5 per cent. synthetic salicylic acid.

Following this line of practice restores the vital conditions at the outset, and reduces to the minimum the dangers of systemic infection, conserving at once both the welfare of the patient and the dentist's conscience and repution.

### SOME ERRONEOUS IDEAS TO BE AVOIDED

(1) That degenerative changes due to regional debility constitute a "low form of inflammation." Inflammation is vitality *plus*, and to confuse the terms regional debility and inflammation would be as misleading as abolishing the minus sign in mathematics and calling it a "low form of plus."

(2) That numerous drugs are required in the treatment of Riggs' disease. Remember the truthful adage that "A multitude of remedies is the child of ignorance."

(3) That you must seek to drive out inflammation. Think it over; it is not there, but must be induced before recovery can ensue.

(4) That any drug is "healing." Nature is the only healer, and we must not obstruct her processes, but seek to aid them.

(5) That our chief duty is to "cast out devils" by infiltration of necrotic tissue with drugs; better far the *vis medicatrix naturae*, the most potent and never harmful agent.

### CONCLUSIONS

*First:* Riggs' disease results primarily from degenerative changes in the investing structures of the teeth.

*Second:* Irritation, as the important secondary cause, would not, with normal vital resistance, result in destruction, but would lead to reaction and repair.

*Third:* The primary cause is the result of inadequate nutrition—regional debility of the deeper structures. Changes in the gingivae are merely symptomatic.

*Fourth:* In induced hyperemia we have a therapeutic agent of great value.

*Fifth:* Scaling is more easily performed, less in extent, and safer to patient, if done after the parts are cleansed and revitalized than before.—May *Cosmos*.

